# Call for action: how to improve use of patient-reported outcomes to guide clinical decision making in rheumatoid arthritis

**DOI:** 10.1007/s00296-018-4005-5

**Published:** 2018-03-21

**Authors:** Bruno Fautrel, Rieke Alten, Bruce Kirkham, Inmaculada de la Torre, Frederick Durand, Jane Barry, Thorsten Holzkaemper, Walid Fakhouri, Peter C. Taylor

**Affiliations:** 1 Institut Pierre Louis d’Epidémiologie et de Santé Publique, Sorbonne Universités, UPMC Universitaire Paris 06, Paris, France; 2 Department of Rheumatology, Pitié-Salpêtrière Hospital, Assistance Publique-Hôpitaux de Paris, Paris, France; 3 Schlosspark-Klinik University Medicine, 14059 Berlin, Germany; 4 Department of Rheumatology, Guys and St Thomas’ NHS Trust, Great Maze Pond, London, SE1 9RT UK; 5 Eli Lilly and Company, Indianapolis, IN USA; 6 Lilly France, Neuilly-sur-Seine, France; 7 Eli Lilly and Company, Basingstoke, Hampshire UK; 8 Lilly Deutschland GmbH, Bad Homburg, Germany; 9 Botnar Research Centre, NDORMS, University of Oxford, Oxford, UK

**Keywords:** Patient-reported outcomes, Rheumatoid arthritis, Pain, Fatigue, Quality of life

## Abstract

Current guidelines for the management of rheumatoid arthritis (RA) recommend early treatment and a treat-to-target goal of remission or low disease activity. Over the past decade, this approach has been extremely successful in reducing disease activity and joint damage in patients with RA. At the same time, however, overall patient perception of well-being appears to have decreased with respect to outcome measures considered important by patients themselves, such as pain, fatigue, physical function and quality of life. The timely and effective use of patient-reported outcomes (PROs) could encourage physicians to focus more on the impact of RA on patients and how patients are feeling. This in turn would facilitate shared decision making between patients and physicians, ultimately leading to a more patient-centered approach and improved patient care. Indeed, PROs provide information about individual patients that complements information provided by physical assessment and composite scores, and can also be used to guide patient care, such as determining whether a clinic visit is needed or whether treatment modifications are necessary. This is particularly important for patients who do not achieve the aspirational target of remission or low disease activity with pharmacological treatment. A number of validated PRO questionnaires are available, but how and which PROs should be incorporated into rheumatology clinical practice as part of the decision-making process is still controversial. Combining PROs with technology, such as computer adaptive tests, electronic PRO systems, web-based platforms and patient dashboards, could further aid PRO integration into daily rheumatology clinical practice.

## Introduction

Current guidelines for the management of patients with rheumatoid arthritis (RA) focus on early intervention and a treat-to-target goal of remission or low disease activity [1, 2]. Over the past decade, this approach has been extremely successful in reducing disease activity and joint damage in patients with RA [3, 4], and patients are now being diagnosed after a shorter symptom duration and with less severe inflammation. Paradoxically, however, these changes have not always been paralleled by an improvement in patient well-being with respect to various patient-reported outcomes (PROs), such as pain, fatigue, morning stiffness and disease activity, possibly due to greater societal and patient health expectations [4].

The challenge now in RA is to encourage rheumatologists and other healthcare professionals (HCPs) to focus on individual patient perceptions of disease impact as well as disease activity measures, with the aims of providing patient-centered care through shared decision making and improving outcomes considered important by patients themselves. This should be achievable using PROs to complement disease activity as a treatment target, and with a view to informing management decisions about appropriate interventions. This is important for all patients, but particularly for individuals who fail to achieve and sustain the target of remission or low disease activity with pharmacotherapy, and those meeting low disease activity or remission states who continue to experience symptoms, such as pain and fatigue, that impact on sense of well-being.

The importance of PROs in patients with RA was highlighted during recent discussions regarding the latest (2016) update to European League Against Rheumatism (EULAR) recommendations for the management of patients with RA [2]. In these discussions, patients suggested that the list of treatment recommendations should end with an item about PROs to convey the importance of PROs in disease management. Fortunately, today, the clinical care of patients with RA increasingly involves patient-centered management by a multidisciplinary team that includes rheumatologists as well as other HCPs, such as general physicians, physiotherapists, occupational therapists and nurses, many of whom are experienced in guiding and steering patient care based on the use of PROs.

The aim of this expert opinion article is to discuss what information can usefully be provided by patient input in the form of PROs to assist in shared decision-making between patients, rheumatologists and other HCPs in daily clinical practice, encouraging a more complete and patient-centered approach to the treatment of patients with RA. This in turn should improve patient care and help achieve optimal health outcomes. This manuscript was developed with the aid of references identified through non-systematic searches of the internet, including PubMed and Google Scholar, using the search terms: ‘patient-reported outcomes’; ‘patient-reported outcome measures’; ‘clinical practice’; ‘rheumatoid arthritis’.

### What are PROs and how are they currently used in the assessment of RA?

Patient-reported outcomes (PROs) are outcomes that focus on the patient’s perspective: they are any reports of a patient’s health status that come directly from the patient [5], and include assessments of symptoms (nature and severity), and patient functioning, health-related quality of life (HRQOL) and satisfaction with current health state. PROs are included in the core set of RA outcome measures recommended for clinical trials by the American College of Rheumatology (ACR), EULAR and Outcome Measures in Rheumatology Clinical Trials (OMERACT) [6–9], and are now mandatory for submissions to drug agencies [5, 10]. PROs provide useful information about the impact of RA on patients’ lives, as well as the effect of an intervention on clinical symptoms in clinical trials and research studies. In this setting, data from PROs are aggregated for group comparisons, and an individual patient’s results are not provided. In addition, the PROs used tend to reflect the requirements of the stakeholders [11]. In real-world clinical practice, however, the need for PROs is different: PROs should provide key information about the individual patient perspective that complements information provided by physical assessment and composite scores, and can also be used to guide patient care [12]. As part of a value-based case, PROs can also be used to shape guidelines [13]. However, the uptake of PROs in daily clinical practice has generally been limited. Although PROs could help to explore the patient perspective and motivations, which is an important consideration in the shared decision-making process, most HCPs focus on objective signs of inflammation, such as tender joint count (TJC) and swollen joint count (SJC) or acute-phase reactants [11, 14–16].

Both ACR and EULAR guidelines for the treatment of patients with RA recommend that treatment is based on a shared decision between the patient and rheumatologist [1, 2]. The benefits of shared decision making include alignment of patient and rheumatologist considerations and aims, improved patient adherence to medication and improved patient satisfaction with management decisions [2, 17]. PROs can aid in the shared decision-making process by presenting patient perspective on the disease impact.

In clinical practice, disease activity is measured using composite indices, such as Disease Activity Score with 28-joint count (DAS28), Clinical Disease Activity Index (CDAI) or Simplified Disease Activity Index (SDAI) [18]. PROs are incorporated into these indices in the form of the Patient’s Global Assessment of Disease Activity (PGA) [19, 20]. The PGA covers two patient-reported concepts: global health and overall disease activity [21]. In its guidelines, EULAR also recommends improvement of disability, HRQOL, and social and work capacity as a treatment target without specifying measurement instruments [2]. OMERACT has also highlighted the concept of patient well-being, measured using the Patient Acceptable Symptom State (PASS). This is a PRO and is defined as a symptom state that the patient considers acceptable. It recognizes that patients consider feeling good to be more important than feeling better [22].

Although PROs are included in composite measures currently used to evaluate patients with RA, they do not fully address the needs of patients for several reasons. In clinical trials, many PROs are used to provide a comprehensive assessment of symptoms, functional status and HRQOL. In clinical practice, however, the number of PROs included in composite measures is limited and they do not reflect aspects of the disease that are important from the patient perspective, such as fatigue, sleep disturbance and psychological aspects [11, 15]. Studies have also shown that many patients in DAS28 remission are not pain-free, suggesting that, in such cases, perceived pain may be non-inflammatory in origin or reflect central sensitization [23, 24]. In addition, composite scores used to assess disease activity do not provide information about disability [11]. The PGA, which is the main PRO in ACR- and EULAR-endorsed instruments for measuring disease activity and patient functioning, is also not without limitations. These include lack of gold-standards for PGA wording/phrasing, assessment period and scoring system; broad concepts that may cause interpretation difficulties; and being affected by patient factors unrelated to RA itself, such as level of education, psychological distress and comorbidities [21]. Furthermore, although the PGA is included in the DAS28, its weighting is low, so its presence does not affect the final DAS28 score to any great extent.

Clearly, it should not be overlooked that treat-to-target strategies provide the best means of addressing inflammation and its consequences in patients with RA; nonetheless, treating-to-target per se may miss opportunities to enhance overall patient well-being through PRO-directed adaptations in disease management. With a treat-to-target approach, many patients will fail to attain low disease activity or remission and in these situations PROs may be particularly useful for guiding treatment decisions, with the aim of improving health outcomes through patient empowerment.

### What are the unmet needs of patients with RA and how could use of PROs address them?

The treat-to-target approach in patients with RA focuses on reducing inflammation to prevent joint damage, physical disability and mortality [1, 2]. However, patients with the disease consider control of pain and fatigue, and maintenance of physical function and HRQOL to be the most important aspects of care [25–29]. A recent literature review of the unmet needs of patients with RA showed that, despite ongoing treatment, many patients still experience significant pain and fatigue, which have a substantial negative impact on HRQOL. Many patients also have mental health symptoms, such as anxiety and depression, that are not being addressed, and may also experience reduced sexual functioning, and social and work participation, which again have a negative impact on HRQOL [28]. Together, these findings suggest that there may be discordance between the needs of patients with RA and current treatment goals. Indeed, a study assessing discordance between patients’ and physicians’ global assessment of disease activity found that nearly 36% of patients experienced discordance from their rheumatologist [26]. However, it should be stressed that optimal management of the inflammatory component of RA, particularly when sustained remission is achievable, may be the best means of addressing the long-term needs of individual patients.

The timely and effective use of PROs could bridge the gap between HCP and patient perspectives in RA by encouraging HCPs to focus more on individual patient perceptions of disease impact. This in turn would facilitate shared decision making between patients and HCPs, encouraging customized and more comprehensive patient care. In addition, PROs may be used to identify flares, and to indicate when a clinic visit is necessary when patients can no longer self-manage their condition. However, PROs will only add value if they inform a management decision that helps to improve patient overall well-being and provide information that rheumatologists and other HCPs would otherwise be unable to capture (e.g. disease activity status between clinic visits), and if obtaining the PRO data and acting on the information obtained do not add to rheumatologist workload. The involvement of a multidisciplinary team in patient care can aid in this. Furthermore, some symptoms, such as fatigue, are not easy to manage, and attempts to do so could prove problematic because this symptom is affected by many factors (e.g. systemic inflammation, comorbidities, sleep quality, physical functioning, mood, pain and sense of control), and interplay between these factors is poorly understood [30]. Currently available PRO instruments could be used to achieve the goals mentioned above. However, additional PROs may be useful to help us understand the extent to which pain is driven by inflammation, and the role of pain and fatigue in causing disability and vice versa.

## Do PROs provide useful additional information beyond rheumatologist assessment to guide therapeutic decisions?

In some instances, PROs are already being used in clinical practice to evaluate patients with RA [31–34], but the question is whether PROs provide added value beyond the information provided by HCP assessment of disease activity alone.

### Self-reported disease activity measures

Examples of patient-reported disease activity measures include the PGA [21], the Routine Assessment of Patient Index Data-3 (RAPID-3) [35] and the Rheumatoid Arthritis Impact of Disease (RAID) instrument [25]. As described earlier, the PGA is incorporated into most of the ACR- and EULAR-endorsed RA assessment tools, and covers two concepts: global health and overall disease activity. It is scored using a numeric rating scale (NRS), a verbally administered NRS, or a visual analog scale (VAS) on a scale of 0–10 cm or 0–100 mm, with higher scores representing higher disease activity or worse global health [21]. RAPID-3 includes the three PROs of pain (VAS), disease activity (PGA VAS) and physical function [multidimensional (MD)-Health Assessment Questionnaire (HAQ)], each scored on a scale of 0–10. Thus, total scores range from 0 to 30, with higher scores indicating greater pain and disease activity, and worse physical function [35]. RAID was developed as a EULAR initiative to combine the most important PROs into one measure [25]. It consists of seven domains covering pain, physical function, fatigue, sleep, physical and emotional well-being, and coping. Each domain is scored using a NRS, giving a total RAID score of 0–10, with higher scores indicating greater disease impact. A RAID score of < 2 is considered to be a PASS [36].

#### What do self-reported disease activity measures add?

Use of self-reported disease activity measures allows a more comprehensive approach to the assessment of patients with RA, beyond objective measures of inflammation and structural damage [21]. RAPID-3 shows a good correlation with the DAS28, CDAI and SDAI, and can also be used to predict structural disease progression [35]. It is also one of the most strongly and extensively validated PRO instruments [37]. RAPID-3 is quicker to use than formal joint counts, and could therefore save time for the rheumatologist. It can also be used by the patient at home, and could therefore be used to assess disease activity (and detect flares) between clinic visits, allowing closer patient monitoring. Other advantages of RAPID-3 are that it highlights patients’ pain and patients’ overall perception of their condition, and it complies with OMERACT recommendations [35].

RAID shows good correlation with PGA, DAS28 and short-form (SF)-36 physical and mental components. It also demonstrates good reliability and sensitivity to change [38], and the ability to discriminate between active and non-active disease [39]. Finally, RAID offers the advantage of encompassing seven different PROs in one measure [25].

### Self-reported RA flare: OMERACT Flare Questionnaire (OFQ) and FLARE-RA questionnaire

The early identification and resolution of flares in patients with RA is important to reduce the risk of joint damage associated with fluctuations in disease activity [40] and thus improve long-term outcomes. However, there is no consensus definition of a flare. The OMERACT Flare Questionnaire (OFQ) was developed to identify and quantify RA flares, a flare being defined by patients and HCPs as a cluster of symptoms of sufficient duration and intensity that cannot be self-managed by the patient and require initiation, change or increase in therapy. The OFQ is a good example of an instrument developed according to recommendations from the US Food and Drug Administration and in collaboration with patients. It consists of questions about pain, physical function, fatigue, stiffness, participation in life activities and self-management over the past week [41, 42].

The FLARE-RA questionnaire was developed in parallel with the OFQ, again with significant patient input, to assess fluctuations in disease activity between visits to the rheumatologist (since fluctuations in disease activity are associated with increased structural damage [40]) and detect RA flares requiring a change in treatment. For this questionnaire, RA flare is defined as any disease exacerbation, either transient or long lasting [43]. The self-administered instrument consists of 11 questions, and the overall score (0–10) is associated with disease activity scores over a 3-month period before the visit to the rheumatologist, even in patients in remission or with low disease activity at the visit [14]. The ability of the FLARE-RA instrument to detect worse outcomes in terms of functional ability or structural damage is under investigation.

#### What do the flare questionnaires add?

Theoretically, the flare questionnaires can capture information not readily available to the rheumatologist, such as a past or present flare based on patient self-report, thus indicating whether a consultation is necessary [42, 43]. However, clinical experience with the flare questionnaires is limited as they have only recently been developed, and their added value in routine clinical practice remains to be determined.

### Health Assessment Questionnaires

The original HAQ is a generic, self-administered questionnaire that measures patient physical functioning through three patient-centered domains of disability, pain and global health [43]. Although it is not specific to RA, the questionnaire is very responsive to change, particularly in patients with high disease activity [44]. The HAQ or one of its many versions [e.g. modified HAQ (MHAQ), MDHAQ, HAQ-II and HAQ-Disability Index (DI)] is one of the most widely used PRO instruments and is endorsed by the ACR and EULAR [1, 18]. As HAQ and HAQ-DI scores predict healthcare resource use [45–47], the HAQ and HAQ-DI also provide useful information for other decision-makers and payers [48–50].

#### What do the HAQs add?

Physical function measured using the HAQ is part of the ACR/EULAR-recommended definition of a good outcome [51]. HAQ or HAQ-DI scores predict comorbidities [52], mortality [53], healthcare resource use (including drug use and the need for joint replacement) [45–47], the need for social support measures [54], the likelihood of employment and productivity loss [46, 55], and costs [46, 56, 57].

### Pain and fatigue

Two types of tools are available for measuring pain in patients with RA: single-item tools that measure pain intensity, such as the VAS, the NRS and the verbal rating scale (sometimes referred to as the Likert scale), and composite measures that encompass several aspects of pain (severity, frequency, duration and location), such as the pain subscales of the Arthritis Impact Measurement Scales (AIMS) and the Medical Outcomes Survey Short Form-36 (SF-36) bodily pain subscale. In clinical practice, single-item tools are useful to provide a measure of overall pain, while composite tools may be useful to address more specific pain issues [58].

The ACR and EULAR do not recommend any specific tools to measure fatigue in patients with RA, but several appropriate instruments are available, including VAS (0–10 or 0–100), the functional assessment of chronic illness therapy-fatigue (FACIT-F), and the SF-36 vitality subscale. FACIT-F covers the physical, functional, emotional and social consequences of fatigue. It consists of 13 items, each scored on a 5-point scale. Scores range from 0 to 52, with higher scores indicating less fatigue [59].

#### What do measurements of fatigue add?

Functional assessment of chronic illness therapy-fatigue (FACIT-F) encompasses various fatigue concepts while providing a global fatigue score [59]. It shows good sensitivity to change, and a significant correlation with TJC, SJC, CDAI, PGA and physician global assessment [59, 60]. In addition, it shows an association with HAQ scores and provides an indication of treatment efficacy [61]. FACIT-F is simple to understand and would be easy to implement in clinical practice [59].

### HRQOL

Patients with RA have significantly impaired HRQOL, and it is now recommended that clinical trials of new treatments for RA include assessments of this parameter [62]. The UK National Institute for Health and Care Excellence also recommends that patients with RA are assessed for the impact of the disease on their lives, including HRQOL [63]. Several different tools are widely used for measuring HRQOL in patients with RA, including the SF-36, the RA Quality of Life Scale (RAQoL), the EuroQoL (EQ)-5D and the Health Utilities Index–Mark 3 (HUI3) [62, 64]. Other country-specific instruments are also available, such as the Musculoskeletal Health Questionnaire developed in the UK by Arthritis Research UK [65]. However, this instrument was developed for use across all musculoskeletal conditions rather than specifically for patients with RA. There is a paucity of information in the literature about the usefulness of measuring HRQOL in clinical practice. A survey conducted at the 2008 EULAR Congress showed that only 51% of rheumatologists used the SF-36 to measure HRQOL and the frequency of assessment was approximately once a year [66].

#### What do HRQOL measurements add?

Health-related quality of life (HRQOL) data provide information about the general mental and physical wellbeing of patients; such data cannot be obtained through disease activity measures. Certain dimensions of the SF-36 (vitality and emotional role functioning) predict remission [67], while a decrease in HRQOL can indicate an increased likelihood of stopping work, being absent from work and reduced productivity, which may have consequences for both patients and society [62].

### Work productivity

Work productivity impairment encompasses both sick leave (absenteeism), which is easily assessed using standard questions on working days missed due to RA, and reduced productivity while at work (presenteeism), which is more difficult to assess. For clinical research in RA, OMERACT recommends five at-work productivity loss measures. These include the Workplace Activity Limitations Scale (WALS), the Work Limitations Questionnaire with modified physical demands scale (WLQ PDmod), the Work Ability Index (WAI), the arthritis-specific Work Productivity Survey (WPS), and the Work Productivity and Activity Impairment (WPAI) questionnaire [68].

#### What do measurements of work productivity add?

The assessment of work productivity provides an indication of the extent of disability, while the WPS also provides information about ability to participate in family, social and leisure activities [69]. The assessment of work productivity also provides an indication of the effectiveness of treatment, since clinical trials of biologic drugs have shown that these agents can reverse reductions in patient productivity and participation due to RA [62].

### Patient-acceptable symptom state

The concept of PASS, which recognizes that it is more important for patients to feel good than to feel better, was highlighted at OMERACT 8 [22]. PASS is a simple question about patient satisfaction with symptom state with regard to pain, PGA and function [70]. At OMERACT 8, the threshold for PASS most frequently chosen by participants was 40 for pain, PGA and function (i.e. patients consider their symptom state acceptable if their pain/PGA/function score is below 40 on a scale of 0–100) [22]. In a recent study, the PASS threshold was calculated as moderate disease activity (score ≤ 22) on the CDAI [70]. An important consideration with PASS is the time frame being assessed, as this can influence the results. At OMERACT 8, most participants felt that the anchor question for PASS should either have no time frame or a short (weeks or a few months) time frame [22].

#### What does PASS add?

PASS provides information about patient satisfaction with disease state and may also facilitate patient–HCP communication [22].

## How could PROs be implemented in clinical practice to inform patient management decisions?

For PROs to be incorporated into clinical practice, they need to fulfill certain criteria, such as being short and simple to understand [71–73], easy to interpret [74], free to use and downloadable from the internet [71], and available in numerous languages [75]. In addition, they should facilitate improved quality of care and the interaction between patient and rheumatologist [76]. Guidelines for implementing PROs in general clinical practice have been published by the International Society for Quality of Life Research (ISOQOL) [77] and by a group of experts in the UK [78], although these guidelines are not specifically for rheumatology clinical practice. ISOQOL guidelines address the following key areas: the goals for PRO data collection; the patients to be assessed; which questionnaire(s) to use, when and how often; how to administer and score the questionnaire; who will see the results; how will any issues arising be addressed; any barriers to PRO use that need to be addressed; and how the value of PRO use will be assessed [77]. Porter et al. [78] identified five key areas: reasons for using the PRO measure; characteristics of the measure; the setting; the feedback system; and any additional support needed.

## How can PROs be combined with new technologies to improve their uptake?

Combining PROs with new technologies can also help to improve PRO uptake in clinical practice. The integration of PRO and electronic health record (EHR) data can facilitate individualized patient care that takes into account the outcomes of greatest importance to patients. In addition, the linkage of PRO and EHR data with treatment guidelines can provide the HCP with feedback, enabling provision of the most appropriate healthcare for individual patients. The following sections provide some examples of how technology has been used to incorporate PROs into daily rheumatology clinical practice.

### Computer-adaptive tests

Computer-adaptive tests (CATs) are computer tests that adapt to each individual’s ability, based on the way the individual responded to previous items. Such tests allow patients to assess several health domains, but through completion of only a few questions [79–81]. Examples of CATs used in rheumatology include the tests developed by PRO Measurement Information System (PROMIS) to measure pain interference, fatigue, physical function, social function and depression [81, 82] and Kids-CAT, used to assess HRQOL in children with RA in Germany [79] (Table 1). Bacalao and colleagues [82] successfully incorporated PROMIS CATs into rheumatology clinic flow, enabling further advancement of the treat-to-target approach. Patients completed the tests on computers in the clinic prior to their scheduled appointment, and the rheumatologist entered test results into EHRs using a standardized flow sheet. Rheumatologists reported that this was not a burden. The greatest challenges to incorporating the tests proved to be patients completing the tests in time for the scheduled appointment and limited access to computers in the clinic [82]. The PROMIS physical function CAT has proved superior or equal to the HAQ and SF-36 with respect to clarity, translatability, importance to patients, reliability and sensitivity to change [81], while pediatricians have reported that Kids-CAT is easy to use and integrate into routine clinical practice, provides valuable information, and facilitates patient HRQOL assessment [79].

**Table 1:** Examples of technologies used to incorporate PROs into rheumatology in daily clinical practice

Location (references)	System	Use	Details
**Computer-adaptive tests**			
PROMIS initiative, US National Institutes of Health, USA [81]	PROMIS ten-item physical function CAT	To measure physical function in patients with RA	Ten-item CAT derived from a 154-item bank Estimated completion time: 1 min [83] Patients can complete test on personal computer in waiting room Test is instantly scored, which would allow results to be shared with patient during consultation
Division of Rheumatology, Northwestern University Feinberg School of Medicine, Chicago, USA [82]	PROMIS CATs for pain, physical function, fatigue, social function and depression	To measure pain, physical function, fatigue, social function and depression in patients with RA	CATs consist of 4–12 tailored items [83] Patients complete tests on computers in rheumatology clinic ahead of scheduled appointment Average completion time for each test ~ 1 min [83] Physician enters test results into the EHR using a standardized flow sheet
Two pediatric clinics at the University Medical Center Schleswig–Holstein, Kiel and Lübeck, Germany [79]	Kids-CAT	To assess HRQOL in children (aged 7–11 years) and adolescents (aged 12–17 years) with RA	Covers six health dimensions: physical well-being, psychological well-being, parent relations, social support and peers, school well-being, chronic illness Patients (who must be able to speak and write in test language) complete test at clinic under supervision of a nurse; results provided to pediatrician before consultation Average completion time at first use: 10.5 min for children, 6.0 min for adolescents; completion time decreases with subsequent use
**Electronic PRO systems**			
Rheumatology Department, Karolinska University Hospital, Stockholm, Sweden [32]	Electronic patient-reported data system linked to the web-enabled Swedish Rheumatology Quality Register (SRQ)	Patient monitoring	Before consultation, patients provide data on general well-being (global health), pain, swollen and tender joints, functional ability (HAQ), HRQOL (EQ-5D) and work ability on the secure SRQ website (which is linked to patients’ electronic health records) using touch-screen computer in waiting room (completion time 10–15 min) Data automatically analyzed and trended, and a summary immediately made available to both patient and physician At consultation, patient and rheumatologist review results together
Rheumatology clinic, Jyväskylä Central Hospital, Jyväskylä, Finland [31]	Electronic patient-reported data system	Patient monitoring	Patients complete health questionnaire (including HAQ, pain and morning stiffness) on touch screen 15 min before scheduled appointment Data analyzed and stored on a central server; results made available to rheumatologist before consultation During consultation, results from previous consultations can be viewed and further data added if necessary
**Web-based platforms**			
Hospitals and rheumatology centers across Norway and Finland [34]	GoTreatIT Web	Remote patient monitoring	Patients report disease status via internet directly into hospital computer system (available PROs include HAQ, MHAQ, MDHAQ, VAS pain and fatigue, RAID and PROMIS20) Data immediately visible to staff at the outpatient clinic, who determine if clinic visit needed
The Netherlands [34]	iMonitor	Remote patient monitoring	Patients report disease status via personal computers, tablets or SmartPhones through PROs chosen from a selection by physician to suit each individual patient (available PROs include HAQ, RAID, RAPID-3 and RADAI5) Rheumatologist alerted if pre-defined thresholds not met, allowing patients with poor disease control to be reviewed remotely [84]
Spain [34]	Andar	Remote patient monitoring	Patients complete RAPID-3 questionnaire, healthcare professionals add clinical and laboratory data, and composite scores are calculated Patients can view results and determine treatment targets; nurses decide if clinic visit needed
France [34]	Sanoïa online health record system	Patient self-monitoring	Patients store and monitor health data using personal computer, tablet or SmartPhone Includes PRO questionnaires, such as RAID, RAPID-3 and HAQ, and treatment trackers, enabling patients to produce reports and graphs Patients decide whether to allow physicians to view data

*CAT* computer-adaptive test, *EHR* electronic health record, *EQ-5D* Euro-QoL-5 dimensions questionnaire, *HAQ* Health Assessment Questionnaire, *HRQOL* health-related quality of life, *MDHAQ* multidimensional HAQ, *MHAQ* modified HAQ, *PRO* patient-reported outcome, *PROMIS* Patient-reported Outcomes Measurement Information System, *RA* rheumatoid arthritis, *RADAI5* 5-item Rheumatoid Arthritis Disease Activity Index, *RAID* Rheumatoid Arthritis Impact of Disease, *RAPID-3* Routine Assessment of Patient Index Data-3, *VAS* visual analogue scale

### Electronic PRO systems

The rheumatology department at Karolinska University Hospital in Sweden uses an electronic patient-reported data system to facilitate patient–rheumatologist interactions and improve the quality of care for patients with RA (Table 1). This system is linked to the web-enabled Swedish Rheumatology Quality Register (SRQ), and makes use of real-time, standardized data in the registry provided by patients and rheumatologists. Before the consultation, patients provide data on general well-being, pain, swollen and tender joints, functional ability, HRQOL and work ability on the secure SRQ website using touch-screen computers in the waiting room. Data are automatically analyzed and trended, and a summary is immediately made available to both the patient and rheumatologist. At the consultation, the patient and rheumatologist review the results together. The system encourages patients to participate in their care, improves communication between patients and rheumatologists, and allows evaluation of the effects of treatment at individual, local, regional and national levels [32].

A similar electronic patient-reported data system is used at the rheumatology clinic at Jyväskylä Central Hospital in Finland (Table 1). This system provides information not previously available from medical records and facilitates focused discussions between the patient and rheumatologist, improves quality of patient care, and leads to improved patient outcomes [31].

Use of electronic PROs in patients with early RA has been shown to significantly improve adherence to RA therapy compared with the use of standard paper-format PROs [85].

### Web-based platforms

Web-based platforms provide opportunities for PRO data to be collected from patients at home before clinic visits [34, 86, 87]; for automatic data analysis and trending [31, 32, 34, 87]; to facilitate focused discussions between patients and rheumatologists [31, 32, 87]; for remote self-monitoring; and for clinical decision support. Indeed, electronic remote self-monitoring through web-based platforms is being used in several European countries for patients with RA (Table 1). These systems can be used through personal computers, tablets and/or SmartPhones, and enable remote self-monitoring of disease activity, potentially leading to early identification of disease flares, identification of patients who require a clinic visit, reduced appointment frequency for patients with low disease activity, and interaction between patients and HCPs outside of clinic visits [34, 88]. For example, GoTreatIT Web is used for remote patient monitoring in hospitals and rheumatology centers across Norway and Finland. Patients use this web-based platform to report their disease status via a number of PROs directly into the hospital computer system. The data are immediately visible to staff at the clinic, who determine whether a clinic visit is necessary [34].

### Patient ‘dashboards’

Relatively recent technologies that facilitate PRO use in clinical practice are patient ‘dashboards’. These are clinical decision support software packages that analyze data from multiple tests and sources for each individual patient, and integrate results onto a single interface (dashboard) in an easy-to-read, easily interpreted format (e.g. graphs) [89, 90]. Data can be trended over time and anomalous results identified [91]. If the system is linked to clinical guidelines, it can be used to aid decision making [90]. Patient dashboards improve patient care and outcomes, clinical efficiency, and rheumatologist adherence to guidelines, and contain healthcare costs [89, 90, 92]. They have already been successfully used in the management of patients with diabetes [91] and chronic or malignant diseases [93], and are being trialed in several hospitals in The Netherlands in patients with early RA through the Computer Assisted Management in Early Rheumatoid Arthritis (CAMERA) trials [94].

## Conclusions and “call for action”

Despite substantial improvements in the care of patients with RA over recent years, the treat-to-target approach has led HCPs to focus on inflammatory disease activity, whereas patients generally consider reduction of pain and fatigue, and maintenance of physical and mental function, to be more important. The timely and effective use of PROs in addition to a treat-to-target approach could facilitate shared decision making between patients and HCPs and, ultimately, improve patient care, as recommended in the 2016 update to the EULAR guidelines for the management of RA. In addition, shared decision making through a multidisciplinary team and involving patients should help to minimize the impact of using PROs in addition to clinical outcomes on the rheumatologist workload. Since digitization and eHealth are an important part of future healthcare, combining PROs with new digital technologies could aid their uptake into clinical practice. We propose that rheumatologists and other members of the HCP team become familiar with PROs that are most relevant to their own practice, such as pain and the HAQ PROs. Adding PRO assessments to the existing core set of treatment targets will help address the need for greater patient involvement in the treatment process for RA, as well as providing additional information when rheumatology visits are infrequent or a long time apart. The PROs of pain, fatigue, and physical and social function have shown reliable and validated results in the research setting, and it is now time to assess the feasibility and added value of incorporating them into daily clinical practice when treating to individual targets in well-designed studies. Further research and a consensus are warranted, and we call for action in this regard, with the goal of working together with RA patients to help achieve the best possible health outcomes.
